# Protocol for an attention-matched randomized controlled trial of 2GETHER: a relationship education and HIV prevention program for young male couples

**DOI:** 10.1186/s13063-022-06457-9

**Published:** 2022-06-20

**Authors:** Michael E. Newcomb, Elissa L. Sarno, Emily Bettin, Adam Conway, James Carey, Christopher Garcia, Ricky Hill, Kyle Jozsa, Gregory Swann, Elizabeth L. Addington, Jody D. Ciolino, Kathryn Macapagal, Judith T. Moskowitz, Brian Mustanski, Sarah W. Whitton

**Affiliations:** 1 Institute for Sexual and Gender Minority Health and Wellbeing, Northwestern University, Chicago, IL USA; 2 Department of Medical Social Sciences, Northwestern University, Chicago, IL USA; 3 Department of Preventive Medicine, Northwestern University, Chicago, IL USA; 4 Department of Psychology, University of Cincinnati, Cincinnati, OH USA

## Abstract

**Background:**

Young men who have sex with men (YMSM) are disproportionately impacted by the HIV epidemic in the USA, and a large number of new infections among YMSM occur in the context of main or primary partnerships. At the same time, healthy romantic relationships promote health and wellbeing by improving social support and encouraging healthy behaviors. Thus, we created 2GETHER: a relationship education and HIV prevention program for young male couples. 2GETHER is delivered face-to-face in a university setting and is composed of two group sessions and two individualized skills coaching sessions. We observed strong support of the feasibility, acceptability, and preliminary efficacy of 2GETHER in a pilot trial.

**Methods:**

We are conducting an attention-matched randomized controlled trial (RCT) to test the efficacy of 2GETHER relative to a control condition based on a well-validated positive affect enhancement program. Enrollment occurred between August 2017 and March 2021 in Chicago and surrounding areas, and we enrolled and randomized 128 dyads (*N* = 256 individuals). Follow-up is ongoing and we will examine primary and secondary behavioral outcomes at 12 months post-intervention, with interim follow-up at 3, 6, and 9 months post-intervention. The primary biomedical outcome is sexually transmitted infection incidence at a 12-month follow-up.

**Discussion:**

2GETHER is innovative in that it places an equal emphasis on relationship skill building and HIV prevention. Thus, the program has the potential to impact numerous health-related outcomes. Despite challenges related to the recruitment of couples and the COVID-19 pandemic, we were able to enroll a robust sample of young male couples with sufficient power to detect effects on study outcomes.

**Trial registration:**

ClinicalTrials.gov NCT03186534.

## Introduction

Men who have sex with men (MSM) are disproportionately impacted by the HIV epidemic in the USA [1]. Furthermore, young MSM (YMSM) have higher HIV incidence than adult and older MSM; the highest rates of new infections occur among YMSM aged 25–34, followed by YMSM aged 13–24. Primary partnerships and serious romantic relationships account for a large proportion of new HIV infections among MSM (35–68%) [2, 3], and this proportion has been estimated to be highest among YMSM (79–84%). At the same time, people in marriages and other committed partnerships consistently report better mental and physical health outcomes than their single peers [4, 5], and these findings have been observed specifically among people in same-sex relationships [6, 7]. Thus, optimizing relationship functioning among YMSM may serve as a critical platform for reducing HIV risk and improving overall health and wellbeing. The purpose of this manuscript is to describe the protocol for a randomized controlled trial (RCT) of 2GETHER, a relationship education and HIV prevention program for young male couples in Chicago [8].

### HIV risk among YMSM in romantic relationships

The fact that such a large proportion of new HIV infections among YMSM occur in the context of main partnerships may seem counterintuitive, but there are multiple factors driving these incident infections [2]. First, many YMSM cease using preventive behaviors (e.g., condoms, pre-exposure prophylaxis [PrEP; a once-daily pill that prevents HIV acquisition]) when they perceive their relationship to have become “serious” [2, 9–11], and this move from a casual to serious relationship occurs particularly quickly among younger MSM [12, 13]. Second, YMSM are more likely to take the receptive role during anal sex when they are in a serious relationship, which carries a higher risk for HIV acquisition than the insertive role [2]. Finally, a large proportion of YMSM who are living with HIV are unaware of their HIV status [1]; YMSM who are unknowingly living with HIV may be entering into relationships, eliminating or reducing their use of protective methods because they perceive their risk of HIV transmission to be low, and exposing their partners to HIV.

Furthermore, male couples often establish “relationship agreements” (i.e., monogamy or non-monogamy arrangements), which detail the conditions under which it is acceptable for each partner to have outside sexual partners [14, 15]. Relationship agreements may optimize sexual satisfaction while minimizing HIV risk when developed effectively and adhered to consistently. However, studies are mixed with regard to the number of male couples who do not have an explicit relationship agreement in place [16], and some have reported that a substantial proportion of couples who do have an agreement disagree about the specific rules of their agreement [8, 17, 18]. Furthermore, male couples commonly report breaks in their relationship agreements or noncompliance with agreement rules [17]. Depending on the specific behaviors that coupled MSM engage in with outside partners, unspecified relationship agreements or breaks in agreement rules may expose both members of the couple to HIV if the couple is not using preventive behaviors within the dyad.

### Couple-based HIV prevention and relationship education

To date, couple-based HIV prevention has largely focused on delivering HIV testing and risk reduction counseling to dyads [19, 20], perhaps most prominently through the CDC-endorsed “Testing Together” protocol [21]. This HIV testing strategy has been tailored specifically for male couples in the USA [22], adapted for administration via videoconference [23], augmented to address substance use in male couples [24], and enhanced with medication adherence counseling for serodiscordant couples [25]. However, Testing Together does not address certain relationship skills that are key to building and maintaining relationship agreements, such as effective communication and conflict resolution. Other domestic and global HIV prevention programs for heterosexual couples have more comprehensively provided relationship education skills to enhance HIV prevention uptake [19, 26, 27].

Couple-based HIV prevention programs may be enhanced by teaching relationship skills more comprehensively; by improving the functioning of the dyad, the couple can more effectively develop plans for behavior change together [20]. Relationship education is a field of intervention development and implementation that aims to provide skills that help couples optimize the health and stability of their relationships [28]. Relationship education uses a preventive approach to promote long-term couple health by teaching relationship skills, with a heavy emphasis on skill building in communication and conflict resolution, as well as discussions of relationship quality and commitment. The efficacy of these programs in improving conflict resolution and global relationship satisfaction is supported by meta-analysis [29].

Thus, the 2GETHER intervention integrates HIV prevention and relationship education to optimize relationship functioning and reduce HIV transmission risk among young male couples. In brief, 2GETHER is a hybrid group- and dyad-level intervention delivered face-to-face, consisting of (a) group sessions focused on didactic skills acquisition and (b) dyadic skills coaching sessions for skill implementation. Core relationship education components include communication skills, conflict resolution, and coping with stress, while sexual health skills include sexual satisfaction, navigating relationship agreements, and biomedical and behavioral prevention and risk reduction. 2GETHER showed evidence of feasibility, acceptability, and preliminary efficacy (i.e., significant reduction in HIV risk behaviors, improvement in relationship functioning) in a non-randomized pilot study in Chicago [8]. 2GETHER is also currently being evaluated in a comparative effectiveness RCT in which the intervention is being delivered online via videoconference to young male couples across the USA, relative to a single session couple-based HIV testing, medication adherence, and risk reduction protocol [30].

### Control condition: positive affect enhancement for couples

To evaluate the efficacy of 2GETHER, we sought an attention-matched, highly active intervention that would benefit YMSM health and wellbeing but would not be linked to sexual risk behavior (i.e., the primary target of the 2GETHER intervention). Positive affect is a central component of mental health and uniquely contributes to emotional wellbeing, independent of the effects of negative affect [31, 32]. Positive affect has been recognized as a key contributor to myriad positive health outcomes [33, 34], including engagement in care, medication adherence, and lower mortality among MSM living with HIV [35, 36].

Recent translational scholarship has sought to leverage these links between affect and health through positive emotion skills training [37, 38]. In the first RCT of a positive emotion intervention for HIV-positive individuals, including a large proportion of MSM (Intervention for those Recently Informed of their Seropositive Status; IRISS), newly diagnosed participants reported better mental health outcomes than controls 15 months post-treatment [38]. IRISS had no significant effect on ART uptake or adherence, substance use, or engagement in high-risk condomless anal sex [38]. A subsequent iteration of IRISS for stimulant-using MSM living with HIV observed a significant increase in the odds of viral suppression and decreased viral load up to 15 months post-treatment; it also observed reduced stimulant use up to 12 months post-treatment [37].

While both of the above trials were highly acceptable to participants and produced positive impacts on health outcomes, neither trial observed any effect of the intervention on sexual behavior [37, 38]. Because 2GETHER’s primary outcomes relate to sexual risk behavior, an iteration of IRISS is an ideal attention-matched control. Control-condition participants still receive the experience of a four-session intervention and are likely to benefit from the skills they learn. However, such benefit will likely be qualitatively distinct from any changes in sexual behavior observed in the active condition. Thus, changes in active-condition participants’ sexual risk behavior will likely be attributable to 2GETHER’s curriculum rather than its delivery.

### Trial objectives and hypotheses

The goal of the current study is to conduct an attention-matched randomized controlled trial (RCT) with a 12-month follow-up to assess the efficacy of 2GETHER in reducing HIV risk relative to a positive affect enhancement program for couples. We are recruiting a sample of young male couples living in Chicago and surrounding areas, who will complete intervention sessions face-to-face in a university setting. We make the following specific hypotheses:

H1: For our primary outcomes, we will observe greater reductions in indicators of HIV risk at 12 months post-intervention among participants randomized to 2GETHER relative to control, including reductions in (1) self-reported condomless anal sex with serodiscordant or unknown status partners and (2) urethral and rectal Chlamydia and Gonorrhea infections. In addition to these primary outcomes measured at the individual level, we anticipate significant improvement in self-reported partner relationship agreement concordance and reductions in the frequency of agreement breaks in 2GETHER relative to control. Finally, we hypothesize that indicators of relationship functioning (e.g., communication, satisfaction) will mediate intervention effects.

H2: With regard to secondary and exploratory outcomes, we will observe greater improvements in various aspects of the HIV continua of prevention and care at 12 months post-intervention among participants randomized to 2GETHER relative to control. For secondary outcomes, we expect to observe improvements in HIV testing uptake (among HIV-negative and unknown status participants) and antiretroviral medication adherence (among HIV-positive participants). Exploratory outcomes will be the uptake of and adherence to pre-exposure prophylaxis (PrEP) among HIV-negative and unknown status participants and reductions in viral load among HIV-positive participants.

## Method

We are conducting an attention-matched RCT to test the efficacy of 2GETHER relative to a control condition based on a well-validated positive affect enhancement program [38]. We selected a highly active and attention-matched control condition that is not theoretically linked to HIV risk behaviors. We aim to randomize 200 dyads (individual *N* = 400) to the 2GETHER intervention or control, and we will examine primary and secondary outcomes at 12 months post-intervention, with interim follow-up at 3, 6, and 9 months post-intervention. There were no restrictions regarding receipt of concomitant care during the trial.

The primary HIV risk behavioral outcome will be the occurrence of condomless anal sex acts with serodiscordant or unknown status partners (all casual sex partners will be considered unknown status), and we will account for the reduced risk of condomless anal sex in the context of PrEP use and undetectable viral load (e.g., condomless sex while one has an undetectable viral load may be considered no risk). The primary biomedical outcome will be STI incidence (i.e., urethral/rectal Chlamydia and Gonorrhea), a robust biological indicator of HIV risk. Primary dyadic outcomes will be partner relationship agreement concordance and agreement breaks. Secondary HIV-related outcomes will be indicators of engagement in the HIV continua of prevention and care, including HIV testing (HIV-negative and unknown status participants) and antiretroviral therapy adherence for HIV-positive participants. Exploratory outcomes will be PrEP uptake and adherence (for HIV-negative participants) and viral load (for HIV-positive participants). We will test for dose effects and decay in effects over time, and we will examine relationship functioning as mediators of change in HIV transmission risk. Unless otherwise noted, all outcomes will be measured at the individual level (not couple-level). This is advantageous because HIV risk may also occur with partners outside the relationship. Also, relationships may dissolve during the follow-up period, so measuring individual-level outcomes allows us to examine the effects of 2GETHER behaviors after relationship dissolution. 

### Study setting

The current study takes place at Northwestern University’s Feinberg School of Medicine, located in Chicago, IL, USA. The study sponsor (Northwestern University) played no part in the study design; collection, management, analysis, and interpretation of data; writing of the report; or decision to submit the report for publication. The study team is housed within a research institute focused on sexual and gender minority health, including numerous studies of HIV prevention and care. Investigators and study team members have substantial expertise with the design and execution of behavioral RCTs for HIV prevention.

### Eligibility criteria

Couples are eligible for the current study based on the following inclusion criteria: (1) both members were assigned male at birth and currently identify as men; (2) both members are at least 18 years old, and at least one member of the dyad is aged 18–29; (3) both partners consider the other a main or primary partner (defined for participants as “…someone you feel committed to above anyone else. This would be someone you call your boyfriend, partner, or significant other”); (4) the couple endorses oral or anal sex with one another in the last 90 days; (5) at least one member of the couple reports having condomless anal sex with a known serodiscordant or unknown serostatus main partner or with any casual sexual partner; (6) both read and speak English at an eighth-grade level or better; (7) both are able to make it to the study site on six separate occasions (one visit to complete the baseline, four visits for intervention sessions, and one final visit for the 12-month follow-up); (8) both partners agree to having intervention sessions audio-recorded for training and analysis. Note that inclusion criterion #7 was loosened after the onset of the COVID-19 pandemic, as described below.

Couples are considered ineligible if study staff ascertain inconsistencies between information provided by participants in the eligibility screener and the baseline assessment, if issues arise that could potentially hinder participation (e.g., language barriers, intoxication), if both individuals are not able to consistently travel to the study site for the duration of the intervention (before the onset of the COVID-19 pandemic), or if there is imminent risk for harm due to intimate partner violence. If either member of the dyad reports intimate partner violence, study staff complete individual, private check-ins to assess their safety and provide resources. If an individual reports that they do not feel safe, they do not continue in the study.

### Recruitment, eligibility screening, and couple confirmation

**Fig. 1:**
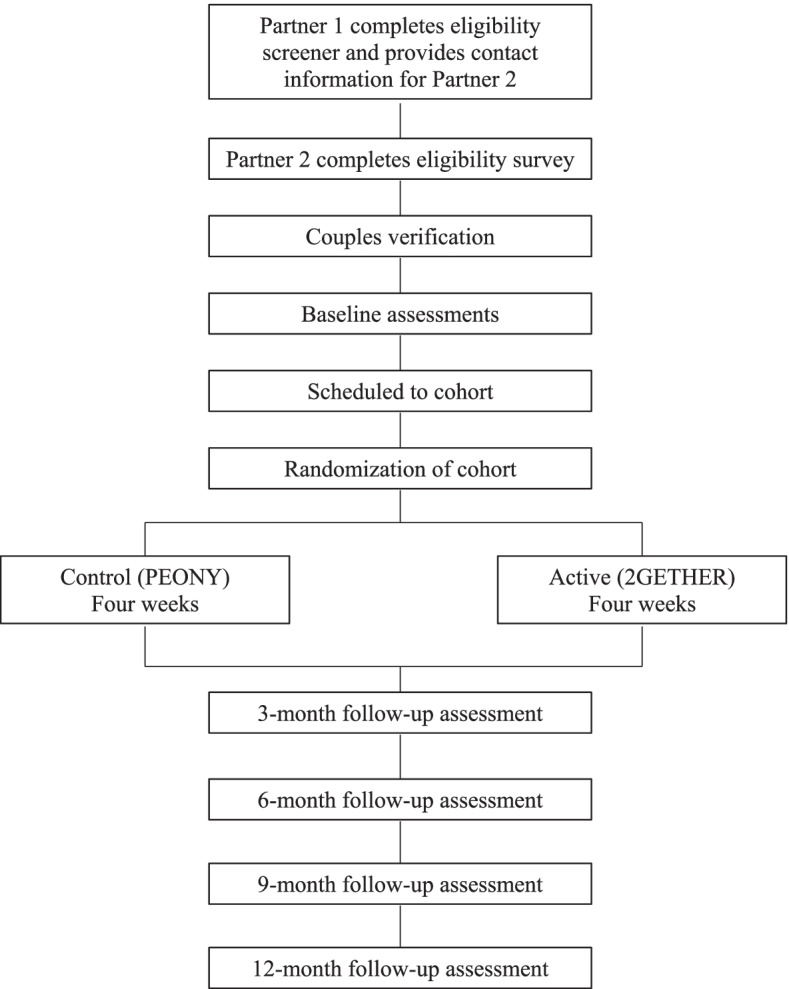
Study flowchart. Measures of primary and secondary outcomes and assessment schedule are displayed in Table 1

Participants are recruited by study staff using in-person Chicago events (e.g., Pride, LGBTQ neighborhood bars), paid advertising on public transit and billboards, flyers posted at community organizations and businesses, paid advertising on social media (e.g., Facebook, Instagram) and geospatial dating/hookup apps, and organic online engagement through social media posts (e.g., Reddit, Twitter). We also partner with a local LGBTQ community-based health organization by embedding research staff throughout their clinic sites to recruit participants for this trial. See Fig. 1 for the flowchart of the study timeline, described below.

All advertisements, events, and posts guide the interested party (i.e., “partner 1”) to a brief online eligibility survey, which includes an infographic illustrating study details, including descriptions of both interventions and tasks required for compensation. Eligibility surveys are administered via REDCap [39]. “Partner 1” is then asked to provide study staff with their partner’s contact information so we may send a confidential link to the eligibility survey for “partner 2.” If “partner 1” would like more time to discuss the study with “partner 2” prior to providing their contact information, they can save and return at a later date; study staff then follow up to answer additional questions and assess interest.

Once both partners complete their respective eligibility surveys, preliminarily eligible couples then complete a couple verification process. The study staff perform two phone conversations, one call with each member of the dyad. During the phone call, staff ask a series of questions to rule out fake couples (e.g., confirm partner demographics, describe couple’s first date); study staff then provide a verbal description of the study to confirm participants’ understanding of study procedures. The couples with consistent responses to couple verification questions then proceed to the baseline assessment.

Baseline assessments are conducted in-person at our offices (adjusted to remote after the onset of the COVID-19 pandemic, as described below). The baseline assessment is a 2-h appointment comprised of completing an online automated informed consent (programmed intro REDCap), a self-report survey, specimen collection for STI testing (i.e., urine and rectal swab), a couple communication task, and for HIV-positive participants, a blood draw for viral load testing. All participants receive $50 for their time at the baseline assessment. At the end of the baseline assessment, the study staff ensure consistency between data provided in the eligibility screener and baseline self-report survey as a final confirmation of eligibility prior to randomization.

### Randomization to the treatment arm

The original plan for randomizing participants was to randomize each couple to either the active or control condition immediately after both members of the dyad completed the baseline assessment using a stratified randomization scheme within blocks of four. After the trial launch in August 2017, we quickly encountered substantial difficulty in randomizing at the couple level; because the first two sessions of the interventions occur in groups, a large enough number of couples would need to complete baseline within the same timeframe, and these couples’ schedules would all need to align in order to convene group sessions. Because of this, we altered the randomization strategy to randomize at the group level. To achieve this, each couple provides their availability for a set of pre-scheduled group sessions occurring over the subsequent 3 months. Once at least two couples sign up for a group session (six couples maximum), all couples within that group are randomized to the active or control condition. All couples must attend their first group session within 3 months of completing their baseline assessment. Participants who do not attend their first session within 3 months are required to re-complete their baseline assessment; otherwise, they are no longer eligible to be enrolled.

To randomize at a group level, we adopted a more flexible covariate-adaptive randomization method known as Minimization [40, 41] on January 16, 2018, after 10 couples had been randomized using the prior approach. Minimization effectively addresses the scheduling difficulties inherent in couple-level block randomization by allowing sequentially recruited clusters of couples (e.g., 4–12 individuals) to be treated as single units, while offsetting imbalance in both individual- and couple-level prognostic variables [42]. Compared to stratification, Minimization can handle a larger number of prognostic factors and is more efficient in controlling imbalance in baseline variables than simple or restricted randomization [43]. In this trial, we set the biasing probability to 0.80 and used the range as the metric of imbalance in the minimization functioning.

Imbalance on the following baseline characteristics is regulated by the allocation algorithm: couple-level age discordance (one partner aged 30 or older), couple-level HIV status (serodiscordant; seroconcordant positive), individual-level STI diagnosis (any positive result), and total number of participants per condition. The couple-level prognostic factors (i.e., HIV status and age discordance) were selected because we hypothesized them to be associated with the primary outcome of HIV risk. By including these factors in the algorithm, we minimize the risk of biased inferences resulting from chance large imbalances in these baseline variables. The individual-level characteristic of baseline STI status was added on October 15, 2018 (after 63 couples had been randomized), to minimize imbalance on this important outcome and outcome-associated variable.

Allocation concealment is protected across all steps of the process. Participants are not considered for randomization until after all baseline activities are complete and their schedules have been matched with a minimum number of other couples. While session dates are determined a priori, intervention arms never are; therefore, session dates cannot be linked to conditions independently. After a group has met all qualifications, the data manager alone is able to trigger the algorithm, which is stored in a secure, restricted, electronic location. Finally, because the biasing probability is *p* = 0.80, a random component is always embedded to prevent deterministic assignment and corresponding selection bias.

### Interventions

#### 2GETHER (active)

2GETHER is comprised of four sessions. First, couples complete two in-person group sessions aimed at skills building, which totals approximately 6.5 h of content. These group sessions address communication skills, coping with stress (both general and sexual minority-specific stress), relationship sexual satisfaction, and HIV transmission risk within the dyad and with outside partners. Two facilitators reinforce core concepts of the intervention through structured conversations about how skills apply to couples’ relationships. In-person groups are attended by 2–6 couples; if a couple does not show up to a group session, we proceed with the session and conduct a make-up session with the missed couple. In rare cases, couples may proceed with the intervention without having completed a make-up session (i.e., if their schedule does not allow), but in these cases, we incorporate missed content into the remaining sessions.

Following the two group sessions, each couple completes two individualized in-person skills coaching sessions (with no other couples attending) with one facilitator, aimed at skills implementation. The first skills coaching session focuses on communication skills and problem-solving. Couples discuss up to two areas of disagreement in session; each partner communicates concerns, actively listens to their partner, and discusses problem-solving, with guidance and corrective feedback from the facilitator to improve the use of these skills. The second skills coaching session, and the zenith of the intervention, focuses on sexual health. Utilizing effective communication skills, couples discuss sexual satisfaction within the dyad, their preferences for a monogamous or non-monogamous relationship agreement, and biomedical and behavioral HIV prevention strategies. HIV-positive participants and HIV-negative participants on PrEP receive medication adherence counseling, based on the Life-Steps protocol [44]. At the end of the sexual health skills coaching session, couples draft a detailed relationship agreement, which includes specific rules about monogamy or non-monogamy and HIV prevention practices. After establishing an agreement, the couple discusses strategies for maintaining or altering the agreement in the future, as well as how they will handle agreement breaks if they occur.

#### PEONY (control)

PEONY (Positive Emotion Orientation for Nurturing Your Relationship) is a positive affect enhancement program based on the IRISS intervention [45], adapted to be administered to couples and to be attention-matched in both format (i.e., hybrid group and dyadic sessions) and time spent in intervention. PEONY group sessions present couples with a didactic overview of positive emotion skills, the function of emotions, and general emotional literacy. Couples then learn and discuss specific positive emotions skills: identifying and capitalizing on positive emotions, gratitude, identifying personal strengths, self-compassion, positive reappraisal, acts of kindness, healthy goal setting, behavioral activation, and practicing mindfulness. Two facilitators reinforce content through structured conversations and guided practice, emphasizing unique opportunities to enact skills as a dyad and benefits to incorporating positive affect skills into relationships.

Following group sessions, couples attend two individualized skills coaching sessions with one of their initial group facilitators; these sessions emphasize skills practice and reinforce previously introduced content. In addition to targeted skills practice, couples participate in exercises designed to promote positive reminiscence of their history as a couple and to highlight positive aspects and specific strengths of their relationships. Participants are also provided with a series of “couple scenarios,” which describe common stressors faced by male couples, and then practice applying specific positive emotion skills that might reduce the negative impact of these stressors.

PEONY teaches tangible positive affect skills with a demonstrated benefit of reducing psychological distress for numerous populations; however, these skills do not address or incorporate the primary mechanisms of change emphasized in the 2GETHER intervention. By design, PEONY does not incorporate or prompt communication or sexual health negotiation skills. Through training, supervision, and fidelity monitoring, PEONY facilitators are further discouraged from the unprompted addressing of couples’ communication and sexual health skills to avoid content contamination across study conditions.

### Facilitator training, fidelity, and supervision

All intervention facilitators hold a bachelor’s degree or higher and have direct experience working in social service or research settings with MSM and/or young adults. Our hiring processes prioritize community-based and direct service experience, including HIV testing and counseling, sexual health education, mental health counseling, workshop facilitation, and program coordination. Employing bachelor-level facilitators with relevant community-based experience (as opposed to licensed mental health professionals) helps to ensure the program is easier to implement in community settings.

All facilitation staff are cross-trained in control and active condition components and are expected to facilitate both conditions. Each facilitator completes an intensive 8-week training protocol, which includes communication skills coaching, HIV risk reduction, medication adherence counseling, and session-specific intervention content for each condition. As part of training, all facilitators complete mock session run-throughs with the principal investigator, co-investigator/supervision lead, and project coordinator for feedback. Facilitators conduct additional mock sessions with professional patient simulators to further develop skills training. Through patient simulation, facilitators gain “real life” session experience where they learn how to lead effective communication practice among dyads and handle challenging or emotionally volatile situations. Additionally, staff participate in periodic “booster” trainings throughout the study to advance their facilitation skills, including motivational interviewing, harm reduction, and mindfulness training.

Facilitators receive weekly supervision through review of their audio-recorded 2GETHER skills coaching sessions. Supervision is principally provided by one of three doctoral-level clinical psychologists and a master-level HIV test counselor in a group setting. During group supervision, pertinent audio segments are played to highlight areas for improvement and reinforce facilitators’ skillful handling of difficult situations. Once facilitators master 2GETHER content, they are invited to provide peer supervision to fellow facilitators. Supervision for the control condition is conducted separately using an analogous format and led by a PhD-level positive emotions project manager and a master-level project coordinator trained in both active and control condition content. Given that this trial uses the same facilitators across conditions, supervision also aims to minimize drift in content across conditions and promote fidelity.

To further ensure fidelity to the intervention protocol and minimize contamination across study conditions, facilitators audio-record all 2GETHER and PEONY sessions. Twenty percent of sessions (both group and individualized couple sessions) are randomly selected for review by an independent assessor to monitor content delivery. All staff members trained in intervention delivery assist with fidelity monitoring. To minimize bias, facilitators are eligible to conduct fidelity assessments only for participants with whom they did not work in sessions. Fidelity monitoring assessors complete a binary checklist indicating whether the essential components of each intervention session were completed effectively by facilitators. In addition, assessors rate facilitator time management, completion of session activities, addressing of participant concerns and questions, participant engagement, and familiarity with session materials and content.

### Study assessments

Study assessments consist of a baseline assessment and follow-up assessments conducted at 3, 6, 9, and 12 months post-intervention. Baseline and 12-month follow-up assessments are conducted in-person, while the interim assessments are conducted remotely online (note changes to in-person assessments due to COVID-19 below). The baseline and 12-month follow-up assessments consist of three elements: a self-report survey hosted on REDCap, STI testing for urethral and rectal Chlamydia and Gonorrhea, and a video-recorded couple communication exercise. The interim assessments at 3, 6, and 9 months consist of a self-report survey only. Participants are compensated $50 for completing each assessment time point, for a total of up to $250 for each member of the dyad. See Table 1 for a list of primary and secondary outcomes by assessment time point.

**Table 1:** Primary and secondary outcomes and assessment schedule

Outcome type	Construct	Measure/operationalization	Measurement schedule				
			Baseline	3 mos	6 mos	9 mos	12 mos
Primary	HIV risk behavior	Condomless anal sex with a serodiscordant main partner or any casual partner [46]	X	X	X	X	X
	STI incidence	Urethral and rectal Chlamydia and Gonorrhea – Aptima Combo 2 GC/CT nucleic acid amplification test [47]	X				X
Secondary: dyadic HIV risk	Relationship agreements	Partner concordance in (non)-monogamy agreement type and rules	X	X	X	X	X
	Agreement breaks	Past 3-month breaks in (non)-monogamy agreement rules	X	X	X	X	X
Secondary: HIV prevention and care continua	HIV/STI testing	Past 3-month HIV and STI testing history	X	X	X	X	X
	PrEP use and adherence	Current and past 3-month PrEP use; adherence over 7, 30, and 90 days [48–50]	X	X	X	X	X
	ART adherence and viral suppression	Adherence over 7, 30, and 90 days [49]; self-reported viral load	X	X	X	X	X
		Lab-collected viral load (baseline and 12 months only)	X				X
Secondary: relationship functioning	Relationship satisfaction	Couples Satisfaction Index – 4-items [51]	X	X	X	X	X
	Communication (self-report)	Communication Skills Test – Positive and Negative scales – Adapted (Jenkins N, Saiz CC: The communication skills test, unpublished)	X	X	X	X	X
	Communication (objective)	10-min recorded communication task [52–54], coded with Interactional Dimensions Coding System [55]	X				X

### Participant retention

All randomized participants complete follow-up assessments at 3, 6, 9, and 12 months post-intervention. For 3-, 6-, and 9-month assessments (which occur remotely), participants are emailed a survey link 1 week prior to their target follow-up date and receive regular survey completion reminders from study staff by phone, email, and text. Participants who do not complete a survey within 6 weeks of their target date do not have their responses recorded for that corresponding follow-up point but are eligible to complete future follow-up assessments unless they withdraw from the study. Analogous procedures are used to schedule and engage participants in the 12-month in-person assessment.

In addition to conducting persistent retention reminders across a variety of media, study staff engage in a multitude of strategies to encourage participant completion of follow-up assessments. All participants receive $50 for each completed follow-up assessment as compensation for their time and to facilitate their ongoing participation. Participants also receive a majority of their retention contacts from the same, central staff member throughout the duration of the study to enhance clarity and build rapport. If a participant becomes disengaged, they may be additionally contacted by one of the facilitators of their previous intervention sessions to encourage re-engagement.

If relationships dissolve during the course of the study, participants are encouraged to continue to complete all remaining follow-up assessments as individuals. When completing as an individual, the couple communication task is eliminated from the 12-month follow-up. Furthermore, single participants do not complete questions in the self-report survey assessing current relationship functioning, but they complete additional questions assessing the impact of their relationship dissolution on their health and wellbeing. If a participant indicates they have a new romantic partner during the follow-up period, that partner is given the opportunity to complete a one-time self-report survey. This new romantic partner is also compensated $50 for their responses and time.

### Protocol changes after trial launch

We made two protocol changes after study launch to address barriers that arose with regard to participant enrollment, scheduling, and safety. Both of these protocol changes were facilitated by integrating eHealth strategies into the study that had previously been developed for an online RCT of 2GETHER, described elsewhere [30]. First, we experienced difficulties with enrollment as a result of many couples having very limited availability for group sessions. To address this, we modified the protocol to allow couples to be randomized as a group of “1” if they were unable to attend the next three scheduled group cohorts after completing baseline. For couples who participate individually (i.e., not in a group), we modified the format of the group sessions in order to reduce fatigue that would result from presenting didactic content to one couple for several hours at a time. Based on the protocol from our online version of 2GETHER [30], we provide couples with all didactic content prior to sessions 1 and 2, in the form of self-paced, narrated videos. Thus, sessions 1 and 2 can instead focus on content review and discussion. Sessions 3 and 4 (i.e., skills practice sessions) were not modified for this protocol change as these were already individualized couple sessions.

Second, the onset of the COVID-19 pandemic in March 2020 spurred us to develop remote protocols for all study activities in order to ensure the safety of our participants and staff. Given that we were already in the process of evaluating an online version of 2GETHER [30], the adaptation of the current protocol was relatively straightforward. In the online version of 2GETHER, all sessions are completed via videoconference (e.g., Zoom). Similar to the protocol described above for couples participating in groups of 1, couples are emailed links to self-paced, narrated videos of the didactic content 1 week prior to each of the group sessions, and group sessions focus on content review and discussion. The protocols for sessions 3 and 4 were not altered for online administration, except that they are now conducted remotely via videoconference. With regard to baseline and 12-month follow-up assessments (other interim time points were already completed remotely), we have made several protocol changes to allow for remote data collection. Participants are sent links to their self-report surveys, hosted via REDCap. Couples are also provided instructions for self-recording their couple communication task via Zoom. Finally, participants are mailed STI testing supplies from Molecular Testing Labs® for self-collection of specimens. Participants ship specimens back to the lab, the lab provides study staff with test results, and study staff communicate results to participants. We experienced a lag between the onset of the pandemic in March and the establishment of an agreement with Molecular Testing Labs® in July 2020. Participants scheduled for a baseline or 12-month assessment during this time period completed all assessment components except for STI testing.

### Analytic plan

#### Power analysis

At the onset of this trial, we aimed to randomize 200 dyads (*N* = 400) to the active or control condition and powered our trial based on that enrollment goal. We experienced various challenges to attaining this recruitment goal (primarily those described in the preceding section related to scheduling couples into group sessions and challenges during the COVID-19 pandemic), so we adjusted our recruitment goal based on a second power analysis. This power analysis was conducted using WebPower in R for a cluster randomized trial with two arms. This method allowed us to account for the similarity between members of the same couple. We assumed a conservative intra-class correlation between members of the same couple of 0.5 for the purpose of calculating power. With an adjusted goal of randomizing 125 dyads (*N* = 250), power for the present study will be 0.89 to detect a medium-sized effect and 0.99 to detect a large effect for the differences between the two study arms.

#### Analyses of primary aims

Imbalances in key stratification variables in baseline data that were not addressed via randomization will be adjusted for in the analyses of treatment effects. Analysis of variance and chi-square tests will be employed to identify those baseline differences on prognostic variables, like age discordance and couple HIV status, the main study outcomes, and demographic variables.

Cochran-Mantel–Haenszel tests of two independent binomial proportions will be used to detect differences in the STI prevalence rates, our primary biological outcome, from baseline to 12-month follow-up. Our main behavioral outcome is condomless anal sex with serodiscordant or unknown status partners. In order to account for the nested nature of dyadic data, we will assess outcome, as well as our secondary outcomes, with multilevel latent growth modeling. Data from all four of our follow-up surveys (3, 6, 9, and 12 months) will be incorporated into those growth models. The treatment condition will be used as a dyad-level predictor within the multilevel framework that will predict differences in intercept and slope terms. This method will not only detect differences in change over time by the treatment condition predicting slopes, but we will also detect differences at each follow-up time point by shifting the intercepts from 3-month to 6-, 9-, and 12-month follow-up.

Indicators of relationship functioning will be tested as mediators of significant differences between the two study arms on the primary outcomes. We will employ Fritz’s [56] approach for testing longitudinal mediation in latent growth models. This is a parallel process approach where the mediator is treated as a lagged effect to maintain the temporal order of events between the mediator and the outcome. A percentile bootstrap test will be used to determine the indirect effect of treatment on the outcome through the lagged mediator.

### Oversight and monitoring

All day-to-day project operations were monitored at weekly study team meetings. Weekly meetings were attended by study investigators, project coordinators, data managers, and research assistants. These meetings focused on recruitment processes, eligibility screening and consent, data management issues, and participant retention at follow-up. As described above, weekly facilitator supervision meetings (attended by study investigators and all program facilitators) focused on issues that arose during group and skills coaching sessions.

This study maintains a Data Safety Monitoring Board, which is composed of three members with expertise in biomedical and behavioral approaches to HIV prevention, research with diverse YMSM, clinical intervention development, and the design and analysis of HIV prevention trials. All three members are clinical psychologists and mid-career research scientists with a history of NIH-funded research. Members of the DSMB perform the following activities: (a) review the research protocol and plans for data and safety monitoring; (b) review progress of the trial, including analysis of data quality and timeliness; subject recruitment, randomization and retention; subject risk versus benefit; and other factors that may affect outcome; (c) review serious adverse event reports, provide commentary, and provide oversight to ensure that reports are relayed to the Institutional Review Board (IRB) and to the Office of Human Research Protections (OHRP), as indicated; (d) review analyses of outcome data of the study and review reports of related studies; (e) determine whether the trial should continue as designed, should be changed, or should be terminated based on the data and make recommendations to the NIH, IRB, and investigators considering conclusion or continuation of the study; (f) review proposed modifications to the study prior to their implementation; (g) protect the confidentiality of the trial data and the results of the monitoring; (h) determine whether and to whom outcome results should be released prior to the reporting of study results; (i) following DSMB meetings, provide the principle investigator with written information concerning their findings.

In addition to self-reported HIV risk behavior, the DSMB monitors HIV (self-reported) and STI incidence throughout the follow-up assessments. Given that the study is only conducting biological testing for STIs at baseline and 12-month follow-up, the DSMB will not be able to make effective decisions about suspending the trial in the event that participants in a given condition experienced an increase in incidence. The study is also collecting self-reported STI incidence at 3-, 6-, and 9-month follow-up assessments and HIV incidence at all waves, and the DSMB will use these reports to monitor trial safety in addition to the 12-month biological testing. Finally, the DSMB is monitoring self-reported intimate partner violence victimization and perpetration in order to assess deleterious impacts on relationship health. The DSMB has opted to meet annually via videoconference throughout the course of the trial. Each of the meetings is divided into three parts. First, there is an open session in which the principal investigator presents the progress of the study and answers questions from members of the DSMB. Second, there is a closed session involving the DSMB members and the study data manager to review trial results to date. Third, there is a final session involving only the DSMB members to discuss the progress of the study and the outcome results, to develop recommendations, and to take votes as necessary.

With regard to adverse events, all study personnel were trained in rigorous procedures regarding monitoring and handling adverse events, including assessing participant safety. Possible adverse events that are unanticipated are brought to the attention of the PI and reported immediately to the IRB. The IRB determines whether it is appropriate to stop the study protocol temporarily or provide suggestions/modifications to the study procedures as necessary. Possible modifications include adding these possible adverse events to the consent form and re-consenting all study participants. The PI is responsible for monitoring participant safety at regularly scheduled research meetings. Given the online nature of follow-up surveys, special procedures are required to identify adverse events. First, the study team instructs participants to report any adverse events to the research team as soon as possible via email or phone. Such a report is then immediately brought to the attention of the PI. Next, each assessment collects self-reported data on the experience of IPV; participants who report IPV (either victimization or perpetration) are flagged for study team follow-up, including a safety assessment and referral to resources. The team also monitors HIV incidence (self-reported) and STI incidence (tested at baseline/12 months and self-reported at 3, 6, and 9 months) for possible increases in incidence.

### Dissemination plan

We will disseminate study findings through a variety of avenues, including peer-reviewed publications, academic meetings, and non-academic media. First, trial results will be reported promptly in a peer-reviewed publication, regardless of study findings. We have already begun to disseminate study findings through peer-reviewed journals; because we are also currently completing another RCT of 2GETHER administered online to couples across the USA [30], we have a large combined baseline sample of YMSM couples that received the same baseline assessment that has been fruitful for analyses of various topics related to couple health [57–60]. Second, we have presented findings using data from the baseline assessment at multiple national and international conferences, and we will present trial results through academic conferences as well. Finally, this trial sits within a larger program of research at the Institute for Sexual and Gender Minority Health and Wellbeing. As we do for many of our studies, we will disseminate our findings in a non-scientific format (e.g., infographics) and post these summaries on our website and social media outlets. The datasets analyzed during the current study and statistical code are available from the corresponding author on reasonable request, as is the full protocol.

## Discussion

Romantic relationships provide numerous benefits to individual health and wellbeing [4–7], particularly those that are characterized by high levels of satisfaction and commitment and low levels of conflict. At the same time, a large proportion of new HIV infections among MSM occur in the context of primary partnerships [2, 3], and this proportion is highest among YMSM. Thus, we created 2GETHER [8], an integrated relationship education and HIV prevention program for young male couples, in order to optimize relationship functioning and reduce HIV transmission.

2GETHER is unique among HIV prevention programs because it places an equal emphasis on relationship skill building and HIV prevention. Given that some YMSM report HIV prevention fatigue and desire programs that address their health and wellbeing more broadly, including relationship education [61], 2GETHER provides YMSM with content they want (i.e., relationship skills) while also delivering skills they need (i.e., HIV prevention). 2GETHER is also novel in that it integrates primary and secondary HIV prevention by enrolling both HIV-positive and HIV-negative individuals (in any arrangement of HIV statuses within dyads). Furthermore, it moves beyond simply advocating for condom use by integrating information about both behavioral and biomedical prevention strategies; the integration of various prevention approaches is critical when working with couples because they are simultaneously trying to build dyadic intimacy and pleasure (e.g., condomless sex) while also preventing HIV/STI within the dyad and with potential outside sexual partners.

Despite a history of published studies on couple-based HIV prevention programs for heterosexual couples, and a smaller number of such interventions for adult male couples [19, 20], 2GETHER was among the very first to developmentally adapt relationship education and couple-based HIV prevention content to the needs of YMSM, and this trial will be one of the first to test the efficacy of couple-based HIV prevention for YMSM in an RCT. As several other couple-based HIV prevention programs for YMSM will complete efficacy trials in the coming years [23, 25, 62–64], including a companion RCT of 2GETHER delivered via videoconference [30], the field will have a strong understanding of the efficacy of this intervention approach, as well as potential differences in efficacy across varied intervention modalities (e.g., face-to-face vs. online) and subpopulations of YMSM (e.g., adolescents vs. young adults, differences by HIV status arrangement).

A notable strength of the current trial of 2GETHER is the use of a highly active, attention-matched control condition, which is a positive affect enhancement program based on the work of Moskowitz and colleagues [45]. This trial design has several advantages. First, the use of a highly active control reduces the likelihood that participants will guess whether they are in the active or control condition, which may reduce bias in self-reported data collection at follow-up time points. Second, the positive affect enhancement program has demonstrated positive effects on various outcomes, so all participants will receive some benefit to their overall wellbeing by participating in this trial. Finally, despite its positive effects on mental health and wellbeing, the positive affect enhancement program has previously observed null effects on HIV-related risk behaviors. Taken together, these various advantages mean that if we observe a difference between conditions in primary outcomes, we can be confident that observed differences are due to 2GETHER content, rather than issues related to the trial design.

We should also note several limitations in the trial design. First, intervention facilitators conduct sessions for both the active and control conditions. Supervision focuses on minimizing drift in condition content, but facilitators may periodically compromise fidelity to a given protocol because they facilitate sessions for both conditions. Second, while recruiting participants from a single city/region enhances our ability to recruit a diverse sample due to engagement with local community-based organizations, the sample is not representative of the general population of coupled YMSM. Related, when recruiting a relatively small population (i.e., YMSM couples) from a single city, likelihood of reaching recruitment saturation is higher. As such, we did not reach our initial goal of recruiting 200 couples. Finally, various challenges arose in executing this trial as a result of the onset of the COVID-19 pandemic. Those challenges that may impact study findings include shifting to an entirely remote protocol for study assessments and intervention sessions, as well as the loss of the ability to collect STI specimens for several months.

Despite these limitations, 2GETHER is a highly innovative and promising approach for improving relationship functioning and reducing HIV risk in young male couples. This RCT will provide important information about the efficacy of couples-based HIV prevention and relationship education for a diverse group of young male couples across the USA. Furthermore, the administration of positive affect enhancement skills via the control condition may provide important preliminary data on the ability of these strategies to improve other health outcomes, including mental health and wellbeing, which may be a promising couple-based intervention strategy for male couples in and of itself or in concert with 2GETHER relationship education and sexual health content.

## Trial status

Recruitment for this trial began in August 2017 and completed in March 2021. In total, we randomized 128 couples (*N* = 256) to either 2GETHER (active condition) or PEONY (control). Follow-up assessments at 3, 6, 9, and 12 months post-intervention are ongoing. We acknowledge that most trial protocols are published prior to completion of enrollment and randomization. However, the onset of the COVID-19 pandemic during the course of recruitment resulted in changes to study protocols (see above) that we wanted to make sure to capture in this manuscript. Given that we were unsure when we would be able to return to our standard, pre-COVID-19 procedures (or whether additional changes would need to be made based on the evolution of the pandemic), we chose to complete this protocol paper after finishing enrollment because we believed it was the best way to most accurately capture all study procedures.

## Data Availability

Not applicable — this is a study protocol and is not reporting results.
